# Supplementary material for: Identification of undetected SARS-CoV-2 infections by clustering of Nucleocapsid antibody trajectories
Source: Nat Commun. 2025 May 14;16:4466. doi: 10.1038/s41467-025-57370-z (PMC12078723; doi:10.1038/s41467-025-57370-z)
Supplement: Supplementary file 1 — Supplementary Information [file 41467_2025_57370_MOESM1_ESM.pdf]

Supplementary files

Identification of undetected SARS-CoV-2 infections by clustering of Nucleocapsid antibody trajectories

Leslie R. Zwerwer*[1,2,3], Tim E. A. Peto#[1,4,5,6], Koen B. Pouwels#[5,7,8], Ann Sarah Walker#[1,5,6], and the COVID-19 Infection Survey team

contribution considered equal

1. Nuffield Department of Medicine, University of Oxford, Oxford, UK
2. Department of Health Sciences, University of Groningen, University Medical Center Groningen, The Netherlands
3. Center for Information Technology, University of Groningen, Groningen, The Netherlands.
4. Department of Infectious Diseases and Microbiology, Oxford University Hospitals NHS Foundation Trust, John Radcliffe Hospital, Oxford, UK
5. The National Institute for Health Research Health Protection Research Unit in Healthcare Associated Infections and Antimicrobial Resistance at the University of Oxford, Oxford, UK
6. The National Institute for Health Research Oxford Biomedical Research Centre, University of Oxford, Oxford, UK
7. Health Economics Research Centre, Nuffield Department of Population Health, University of Oxford, Oxford, UK
8. Nuffield Department of Primary Care Health Sciences, University of Oxford, Oxford, UK

*Corresponding author: Leslie R. Zwerwer, Department of Health Sciences, University of Groningen, University Medical Center Groningen, Hanzeplein 1, 9713 GZ, Groningen, The Netherlands. Email: l.r.zwerwer@rug.nl.

Table of Contents

Supplementary File 1. *Supplementary Methods* 3

Supplementary Table 1. *Characteristics of participants with <4 vs ≥4 N-antibody measurements* 4

Supplementary Table 2. *Distributions of differences between estimated infection dates from N-antibody measurements and swab-positive infections.* 5

Supplementary Table 3. *Percentage of participants with estimated N-antibody (hypothetical) infection date within 15, 30, 60, 90, 120 and 180 days of the closest swab-positive infection.* 5

Supplementary Table 4. *Subgroup analysis by vaccination status and epoch on the number of true infections.* 6

Supplementary Table 5. *Classifications and counts of categories for logistic regression model.* 7

Supplementary Table 6. *Associations between characteristics and N-antibody non-response (vs. response) in 17,315 swab-positive infections* 8

Supplementary Table 7. *Seropositivity during the participant's study period from N-antibody trajectory-based analysis, the fixed 30 ng/mL threshold, the fourfold-based antibody classification and the sensitivity analysis on the fourfold-based N-antibody classification and different data sources for swab positivity.* 9

Supplementary Figure 1. *Number of participants providing blood samples in the survey per month.* 10

Supplementary Figure 2. *Study flow chart for the clustering of N-antibody trajectories in participants with any N-antibody measurement.* 11

Supplementary Figure 3. *N-antibody trajectories for 13 clusters identified from K-means clustering with (a) identity transformations, (b) log2 transformation.* 12

Supplementary Figure 4. *Percentage of each cluster by* swab-positivity *infection status, with (a) identity transformations, (b) log2 transformation.* 13

Supplementary Figure 5. *Percentages of each swab-positive infection group in each cluster, with (a) identity transformations, (b) log2 transformation.* 14

Supplementary Figure 6. *Confusion matrix and trajectories for the N-antibody id and log2 clustering classifications.* 15

Supplementary Figure 7. *Reclassified N-antibody trajectories.* 16

Supplementary Figure 8. *Distribution of days between the N-antibody (hypothetical) infection date and closest swab-positive infection.* 18

Supplementary Figure 9. *Flow chart for logistic regression.* 19

Supplementary Figure 10. *Visualisation of N-antibody trajectories by trajectory-based and threshold-based N-antibody classification* 20

Supplementary Figure 11. *Visualisation of N-antibody trajectories by trajectory-based and main fourfold-based classification* 21

Supplementary Figure 12. *Visualisation of N-antibody trajectories by trajectory-based and fourfold-based classification (sensitivity analysis)* 22

References 23

Supplementary File 1. *Supplementary Methods*

PCR testing

Combined nose and throat swabs were tested at high-throughput national "Lighthouse" laboratories in Glasgow (from 16 August 2020 to the end of the survey) and Milton Keynes (from 26 April 2020 to 8 February 2021). The presence of three SARS-CoV-2 genes (ORF1ab, nucleocapsid protein (N), and spike protein (S)) was identified using real-time PCR with the TaqPath RT-PCR COVID-19 kit (Thermo Fisher Scientific). PCR outputs were analysed using UgenTec Fast Finder 3.300.5 (TaqMan 2019-nCoV Assay Kit V2 UK NHS ABI 7500 v2.1; UgenTec), with an assay-specific algorithm and decision mechanism that allows conversion of amplification assay raw data into test results with minimal manual intervention. Samples were called positive if at least a single N and/or ORF1ab gene were detected, and PCR traces exhibited an appropriate morphology. Detection of the S gene alone was not considered to be a reliable positive[1].

K-means clustering of longitudinal N-antibody trajectories

To cluster similar N-antibody trajectories together, we used a longitudinal variation on K-means[2-4]. Similarly to traditional K-means, the algorithm aims to partition participants into a pre-specified number of different clusters (*K*). The algorithm starts with calculating the distance between all longitudinal trajectories and each of the *K* probabilistically initialised cluster centroids (initialisation using K-means++, see[5]) based on a pre-specified distance metric. We used a dynamic time warping distance which calculates the minimal distance between two time series by locally stretching and compressing the time-series in such a way that they are aligned in the most optimal way[2-4]. An important advantage of a dynamic time warping distance function is its ability to calculate the distance between two time series with varying lengths and with different intervals, e.g. due to missed visits, failed assays or end of study participation. Subsequently, each trajectory is assigned to a cluster based on the minimal dynamic time warping distance to each cluster centroid. Next to differences in shape, this distance considers differences in height. Then, the algorithm calculates the updated cluster centroids, here using Barry Center Averages which iteratively creates an average trajectory that minimises the squared dynamic time warping distance to each trajectory in the respective cluster[3]. This process is reiterated until the clustering process converged or the maximum number of iteration is reached (whichever comes first). We performed the analysis using Python 3.10.12 together with the tslearn package (version 0.5.2)[2].

Supplementary Table 1. *Characteristics of participants with <4 vs ≥4 N-antibody measurements*

		**< 4 measurements**	**≥4 measurements**	**Standardized differences (%)**
**Number of participants**		85,040	185,646	NA
**Number of N-antibody measurements (median (IQR))**		2 [1, 3]	7 [6, 8]	NA
**Age at last birthday (years) (median [IQR], percentiles [1, 99])**		49 [33, 64], [10, 86]	57 [44, 68], [18, 84]	NA
**Sex (%)**	Female	45,179 (53.1)	101,644 (54.8)	-0.03
	Male	39,861 (46.9)	84,002 (45.2)	
**Ethnicity (%)**	Non-White	6,929 (8.1)	9,362 (5.0)	0.13
	White	78,111 (91.9)	176,284 (95.0)	
**Long-term health condition (%)**	No	63,702 (74.9)	135,934 (73.2)	0.04
	Yes	21,338 (25.1)	49,712 (26.8)	
**Healthcare worker (%)**	No	81,028 (95.3)	176,087 (94.9)	0.02
	Yes	4,012 (4.7)	9,559 (5.1)	
**Vaccination* (%)**	not vaccinated	12,918 (15.2)	2,474 (1.3)	**0.52**
	1 vaccination	17,277 (20.3)	2,518 (1.4)	**0.64**
	2 vaccinations	38,031 (44.7)	38,799 (20.9)	**0.52**
	3 vaccinations	16,742 (19.7)	141,596 (76.3)	**-1.37**
	4 vaccinations	13 (0.0)	203 (0.1)	-0.04
	Missing	59 (0.1)	56 (0.0)	0.02
**Swab-positive infections† (%)**	No infection	71,300 (83.8)	148,363 (79.9)	0.10
	Infection before the study period	10,689 (12.6) ‡	12,231 (6.6)§	**0.20**
	Infection during the study period	2,877 (3.4)***	23,959 (12.9)#	**-0.35**
	Infection before and during the study period	174 (0.2) ‡***	1,093 (0.6)§#	-0.06
**Spike-antibody seropositivity****	No spike seropositivity	82,099 (96.5)	180,414 (97.2)	-0.04
	Spike seropositive before the study period	2,800 (3.3)	4,646 (2.5)	0.05
	Spike seropositive during the study period	141 (0.2)	586 (0.3)	-0.03

*Vaccination status at the end of each participant's study period.
† As identified from swab test results (see Methods)
** Before any reported vaccinations
‡ 206 participants had two or more swab-positive infections before their study period.
*** 13 participants had two or more swab-positive infections during their study period.
§ 108 participants had two or more swab-positive infections before their study period.
# 350 participants had two or more swab-positive infections during their study period.
IQR: Inter quartile range.
Note: study period defined as the time from each participant's first N-antibody measurement to their last N-antibody measurement. Participants could be in the survey before this started. Considering absolute standardized differences of 0.20-0.8 as modest and >0.8 as large. Bold font represents absolute standardized differences of ≥0.20.

Supplementary Table 2. *Distributions of differences between estimated infection dates from N-antibody measurements and swab-positive infections.*
Note: For N-antibody (hypothetical) infections, infection date was estimated as 14 days before the midpoint between the two measurements with the greatest increase between them. The table includes all individuals with ≥4 N-antibody measurements, an increasing or de- and increasing N-antibody trajectory (identified with the clustering) and a swab-positive infection during their study period (N=18,128).

Statistic	Value
Number of observations	18,128
Minimum	-258.5
5th percentile	-28
First quartile	-4
Median	6.5
Mean	5.1
Third quartile	16.5
95th percentile	36.5
Maximum	280

Supplementary Table 3. *Percentage of participants with estimated N-antibody (hypothetical) infection date within 15, 30, 60, 90, 120 and 180 days of the closest swab-positive infection.*
Note: For N-antibody (hypothetical) infections, infection date was estimated as 14 days before the midpoint between the two measurements with the greatest increase between them. The table includes all individuals with more ≥4 N-antibody measurements, an increasing or de- and increasing N-antibody trajectory (identified with the clustering) and a swab-positive infection during their study period (N=18,128).

Maximum distance from swab-positive infection	
15 days (%)	61.5
30 days (%)	87.6
60 days (%)	97.3
90 days (%)	98.8
120 days (%)	99.3
180 days (%)	99.9

Supplementary Table 4. *Subgroup analysis by vaccination status and epoch on the number of true infections.* The vaccination status was missing for 14 participants with an infection detected by either method, these participants have been excluded from the subgroup analysis for vaccination.

	Subgroup	Number of swab-positives	Number of N-antibody positives	Detected by both methods	Total detected infections	Estimated number of true infections (95%CI)	Undetected infections (%; 95%CI)
Vaccination status	Not vaccinated	1,137	1,294	894	1,537	1,645 (1,619–1,674)	6.6 (5.1–8.2)
	1 vaccination	1,444	1,614	1,054	2,004	2,210 (2,172–2,253)	9.3 (7.7–11.1)
	2 vaccinations > 6 months ago	3,545	3,501	2,558	4,488	4,851 (4,803–4,904)	7.5 (6.6–8.5)
	2 vaccinations 3 – 6 months ago	10,153	10,346	8,012	12,487	13,110 (13,051–13,172)	4.8 (4.3–5.2)
	2 vaccinations ≤ 3 months ago	3,059	3,178	2,218	4,019	4,382 (4,333–4,436)	8.3 (7.2–9.4)
	3 or 4 vaccinations	6,058	4,496	3,387	7,167	8,041 (7,956–8,131)	10.9 (9.9–11.9)
Total		25,396	24,429	18,123	31,702	34,239	7.4
Epoch	Alpha	149	344	46	447	1,109 (905–1,414)	59.7 (50.6–68.4)
	Delta	15,822	17,285	12,510	20,597	21,860 (21,777–21,954)	5.8 (5.4–6.2)
	BA.1	9,433	6,811	5,572	10,672	11,530 (11,453–11,611)	7.4 (6.8–8.1)
Total		25,404	24,440	18,128	31,716	34,499	8.1

Supplementary Table 5. *Classifications and counts of categories for logistic regression model.*

**N-antibody**	**Swab**	**Classification**	**N**
Increasing	During	Correct (reference)	11,603
Increasing	Before and during	Correct (reference)	384
Decreasing and increasing	Before and during	Correct (reference)	129
Decreasing and increasing	During	Correct† (reference)	413
Decreasing	Before and during	Missed infection by N-antibody	150
Decreasing	During	Missed infection by N-antibody	207
Flat	Before and during	Missed infection by N-antibody	74
Flat	During	Missed infection by N-antibody	2,788
All 10	Before and during	Missed infection by N-antibody	33
All 10	During	Missed infection by N-antibody	1,523
All 200	Before and during	Missed infection by N-antibody	2
All 200	During	Missed infection by N-antibody	9

† prior infection from N-antibody may have been before survey participation started.

Supplementary Table 6. *Associations between characteristics and N-antibody non-response (vs. response) in 17,315 swab-positive infections*
Comparing infections detected by both swab positivity and N-antibody trajectory-based analysis (i.e. responders, reference) to infections that were only detected by swab (i.e. non-responders) in 17,315 swab-positive infections from 17,181 participants (7,841 males and 9,340 females). P-values were obtained using a two-sided Wald-test. No adjustments were made for multiple comparisons.

	Univariate			*Multivariate*		
Factors	*Odds ratios*	*95% CI*	*P*	*Odds ratios*	*95% CI*	*P*
Age ≤30y: per 10 years*	1.26	1.08 – 1.48	**0.004**	1.12	0.94 – 1.34	0.202
Age 30–60y: per 10 years*	0.92	0.88 – 0.95	**1.74e-5**	0.87	0.83 – 0.91	**3.39e-10**
Age ≥60y: per 10 years*	1.15	1.05 – 1.25	**0.001**	1.04	0.95 – 1.14	0.399
Female (ref)	1.00	NA	NA	1.00	NA	NA
Male	0.97	0.91 – 1.04	0.415	1.00	0.93 – 1.08	0.953
White ethnicity (ref)	1.00	NA	NA	1.00	NA	NA
Non-White ethnicity	0.83	0.71 – 0.96	**0.016**	0.74	0.63 – 0.87	**2.93e-4**
No healthcare worker (ref)	1.00	NA	NA	1.00	NA	NA
Healthcare worker	1.23	1.07 – 1.40	**0.003**	1.05	0.91 – 1.22	0.481
No long-term health condition (ref)	1.00	NA	NA	1.00	NA	NA
Long-term health condition	1.05	0.97 – 1.14	0.230	1.06	0.97 – 1.16	0.180
Not vaccinated	0.65	0.52 – 0.81	**1.27e-4**	0.53	0.42 – 0.67	**1.23e-7**
1 vaccination	0.89	0.74 – 1.07	0.226	0.80	0.65 – 0.97	**0.025**
2 vaccinations > 6 months ago	1.12	0.98 – 1.28	0.105	0.78	0.67 – 0.91	**0.001**
2 vaccinations 3 – 6 months ago	0.74	0.66 – 0.83	**6.00e-7**	0.64	0.56 – 0.72	**1.10e-12**
2 vaccinations ≤ 3 months ago (ref)	1.00	NA	NA	1.00	NA	NA
3 or 4 vaccinations	2.61	2.32 – 2.93	**< 2e-16**	1.24	1.06 – 1.45	**0.008**
Ct value ≤ 20: per unit higher[†]	1.09	1.07 – 1.11	**<2e-16**	1.02	1.00 – 1.04	0.090
Ct value 20 – 30: per unit higher [†]	0.97	0.95 – 0.98	**3.46e-7**	0.99	0.97 – 1.00	0.078
Ct value >30: per unit higher [†]	1.27	1.22 – 1.33	**<2e-16**	1.33	1.27 – 1.40	**<2e-16**
No symptoms reported (ref)	1.00	NA	NA	1.00	NA	NA
Loss of taste or smell (with or without other symptoms)	0.49	0.44 – 0.54	**<2e-16**	0.65	0.58 – 0.73	**1.17e-13**
Fever or cough (with or without other symptoms (but not loss of taste or smell)	0.98	0.89 – 1.08	0.674	0.83	0.74 – 0.92	**0.001**
Other symptoms only	0.70	0.63 – 0.78	**2.51e-11**	0.60	0.54 – 0.67	**<2e-16**
Delta epoch (ref)	1.00	NA	NA	1.00	NA	NA
BA.1 epoch	3.46	3.24 – 3.71	**<2e-16**	2.76	2.49 – 3.06	**<2e-16**

* Heterogeneity tests for effects of: age ≤30 vs. age 30 to 60 p=0.009, age ≤ 30 vs. age ≥60 p=0.002.
[†] Heterogeneity tests for effects of: Ct value ≤ 20 vs. 20 to 30 p=0.05. Ct value ≤ 20 vs. ≥30 p<2e-16. Ref: Reference, Ct: Cycle thresholds. Bold font indicates statistically significant coefficient (P<0.05).

Supplementary Table 7. *Seropositivity during the participant's study period from N-antibody trajectory-based analysis, the fixed 30 ng/mL threshold, the fourfold-based antibody classification and the sensitivity analysis on the fourfold-based N-antibody classification and different data sources for swab positivity.*

Note: The very small number of second and third swab-positive episodes during the study period were considered to be detected by swab-only, as methods based on N-antibody classifications did not attempt to identify multiple infections within the study period.

		Trajectory-based classification	Threshold-based classification	Fourfold-based classification	Fourfold-based classification (sensitivity analysis)
Survey	Both swab and seropositive	7,227 (3.9)	7,319 (3.9)	6,498 (3.5)	6,759 (3.6)
	No evidence of infection during	159,607 (86.0)	151,437 (81.6)	162,319 (87.4)	160,418 (86.4)
	Only seropositive	17,213 (9.3)	25,383 (13.7)	14,501 (7.8)	16,402 (8.8)
	Swab only	1,629 (0.9)	1,537 (0.8)	2,358 (1.3)	2,097 (1.1)
Survey + NTP	Both swab and seropositive	16,457 (8.9)	16,859 (9.1)	14,857 (8.0)	15,435 (8.3)
	No evidence of infection during	155,410 (83.6)	147,550 (79.4)	157,251 (84.6)	155,667 (83.7)
	Only seropositive	7,983 (4.3)	15,843 (8.5)	6,142 (3.3)	7,726 (4.2)
	Swab only	6,050 (3.3)	5,648 (3.0)	7,650 (4.1)	7,072 (3.8)
Survey + NTP + Self	Both swab and seropositive	18,128 (9.7)	18,595 (10.0)	16,356 (8.8)	16,988 (9.1)
	No evidence of infection during	154,282 (82.9)	146,487 (78.8)	155,951 (83.8)	154,421 (83.0)
	Only seropositive	6,312 (3.4)	14,107 (7.6)	4,643 (2.5)	6,173 (3.3)
	Swab only	7,276 (3.9)	6,809 (3.7)	9,048 (4.9)	8,416 (4.5)
Survey + NTP + Self + Think	Both swab and seropositive	19,257 (10.3)	19,801 (10.6)	17,371 (9.3)	18,057 (9.7)
	No evidence of infection during	152,742 (82.1)	145,024 (77.9)	154,297 (82.9)	152,821 (82.1)
	Only seropositive	5,183 (2.8)	12,901 (6.9)	3,628 (1.9)	5,104 (2.7)
	Swab only	8,961 (4.8)	8,417 (4.5)	10,847 (5.8)	10,161 (5.5)

Survey: only using positive and negative swab PCR test results from the COVID-19 Infection Survey to define swab-positive infections; NTP: using swab positives from national testing programmes in England or Wales (PCR or LFT); Self: self-reported positive swab test results; Think: reports on thinking one had had COVID-19.

Supplementary Figure 1. *Number of participants providing blood samples in the survey per month.*

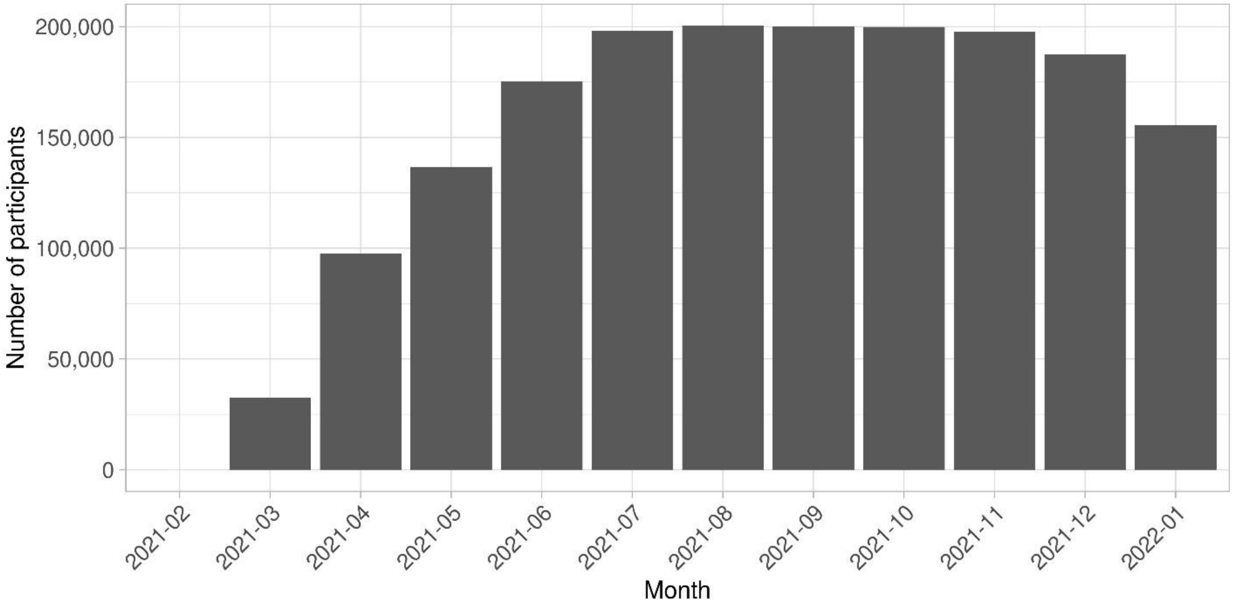

Note: the numbers of participants providing blood samples was increased from March through June 2021 to monitor vaccination responses, see Methods.

Supplementary Figure 2. *Study flow chart for the clustering of N-antibody trajectories in participants with any N-antibody measurement.*

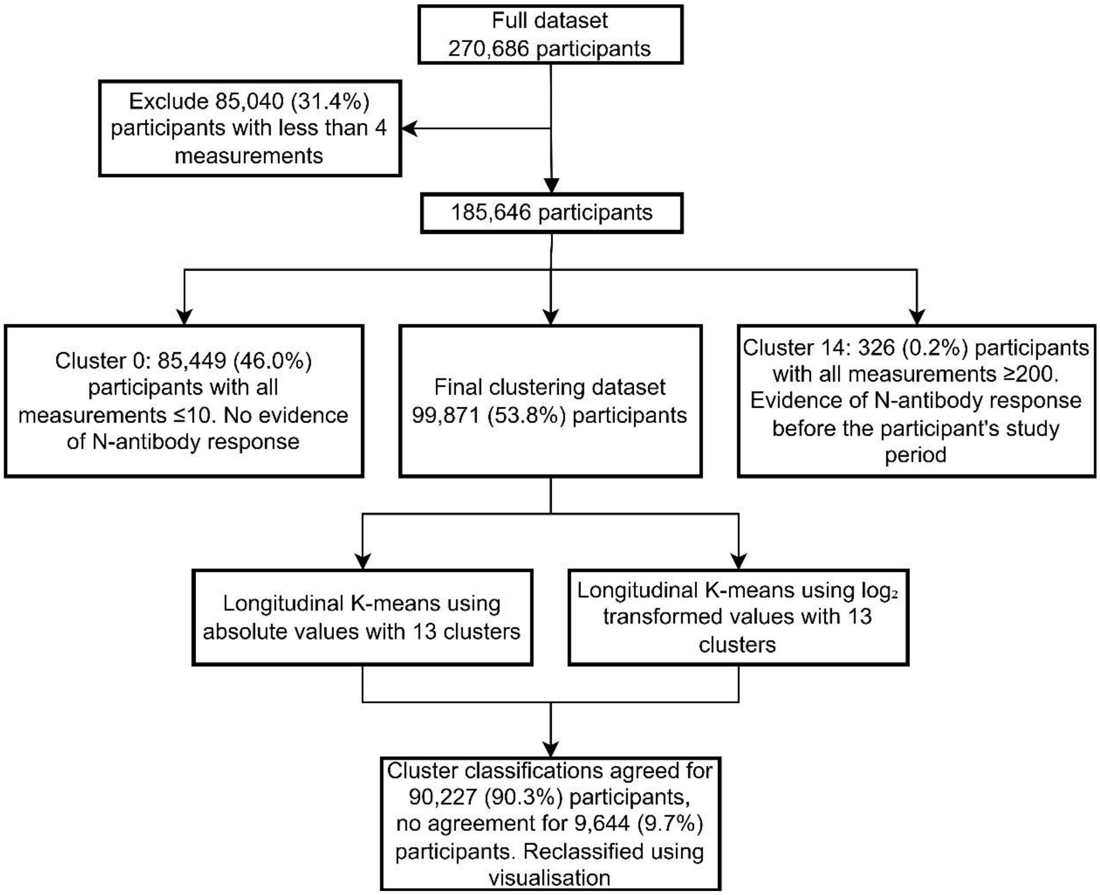

Supplementary Figure 3. *N-antibody trajectories for 13 clusters identified from K-means clustering with (a) identity transformations, (b) log2 transformation.*
Cluster numbers are non-informative and are ordered by cluster classification. Black line depicts a generalised additive modelling smooth for all N-antibody measurements assayed between the 10th and 90th percentile of the centered days in each cluster. Within cluster classifications, clusters are respectively ordered on the degree of flatness, and the amount of decrease and the amount of increase in the generalised additive model smooth line. N-antibody classifications in (a) cluster 0: All N-antibody levels ≤10, clusters 1-3: flat, clusters 4-8: decreasing, clusters 9-12: increasing, cluster 13: decreasing and increasing, and cluster 14: all N-antibody levels ≥ 200; (b) cluster 0: all N-antibody levels ≤10, clusters 1-5: flat, clusters 6-8: decreasing, clusters 9-12: increasing, cluster 13: decreasing and increasing, cluster 14: all N-antibody levels≥ 200. Note that the same participants may be classified in different clusters for the two different transformations. For comparability, trajectories are centered on the midpoint between the maximum difference between any two consecutive measurements per participant.

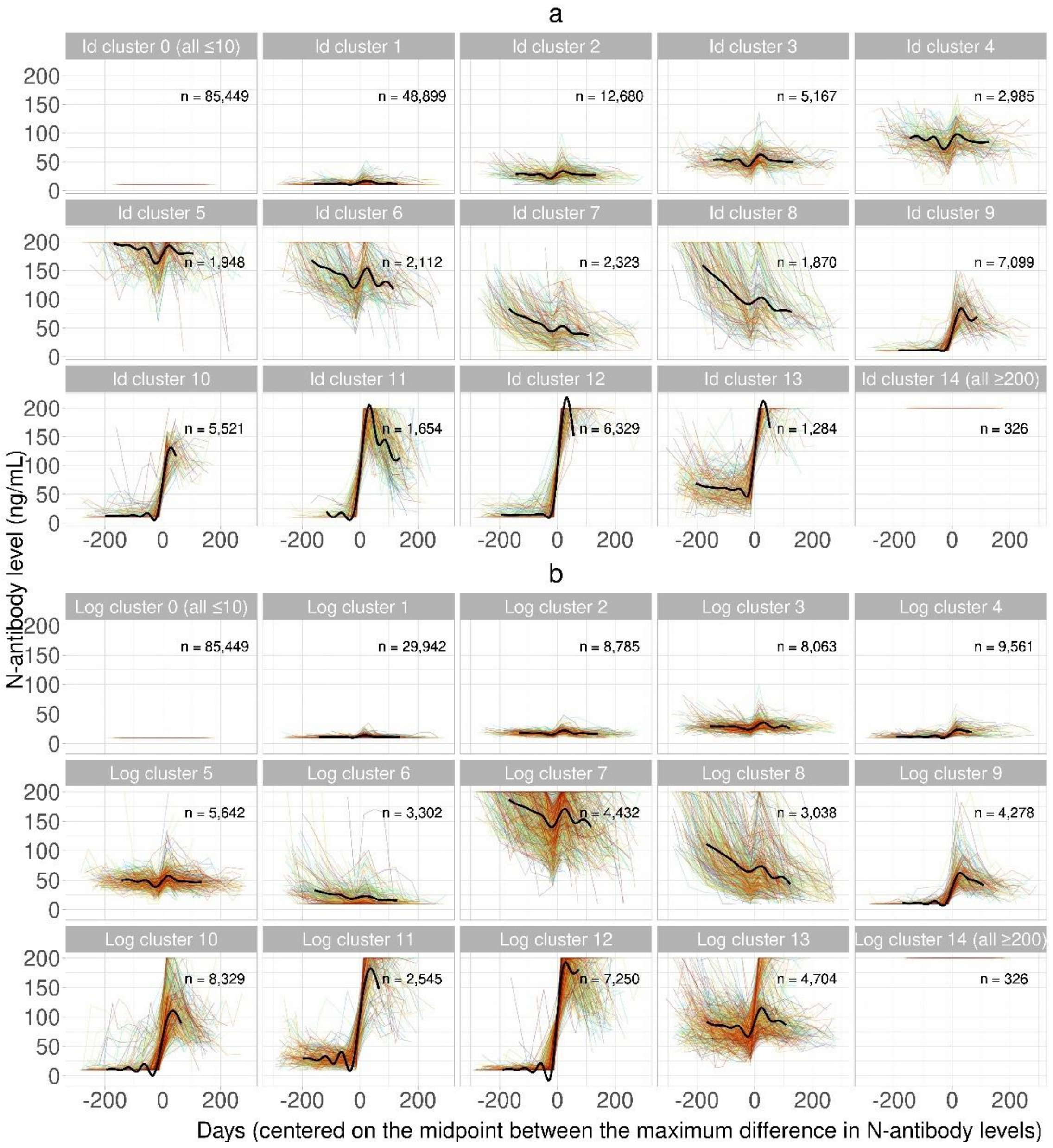

Supplementary Figure 4. *Percentage of each cluster by swab-positivity infection status, with (a) identity transformations, (b) log2 transformation.* Cluster numbers are non-informative and correspond to the cluster numbers in Supplementary Figure 3. N-antibody classifications in (a) cluster 0: All N-antibody levels ≤10, clusters 1-3: flat, clusters 4-8: decreasing, clusters 9-12: increasing, cluster 13: decreasing and increasing, and cluster 14: all N-antibody levels ≥ 200; (b) cluster 0: all N-antibody levels ≤10, clusters 1-5: flat, clusters 6-8: decreasing, clusters 9-12: increasing, cluster 13: decreasing and increasing, cluster 14: all N-antibody levels≥ 200. Note that the same participants may be classified in different clusters for the two different transformations.

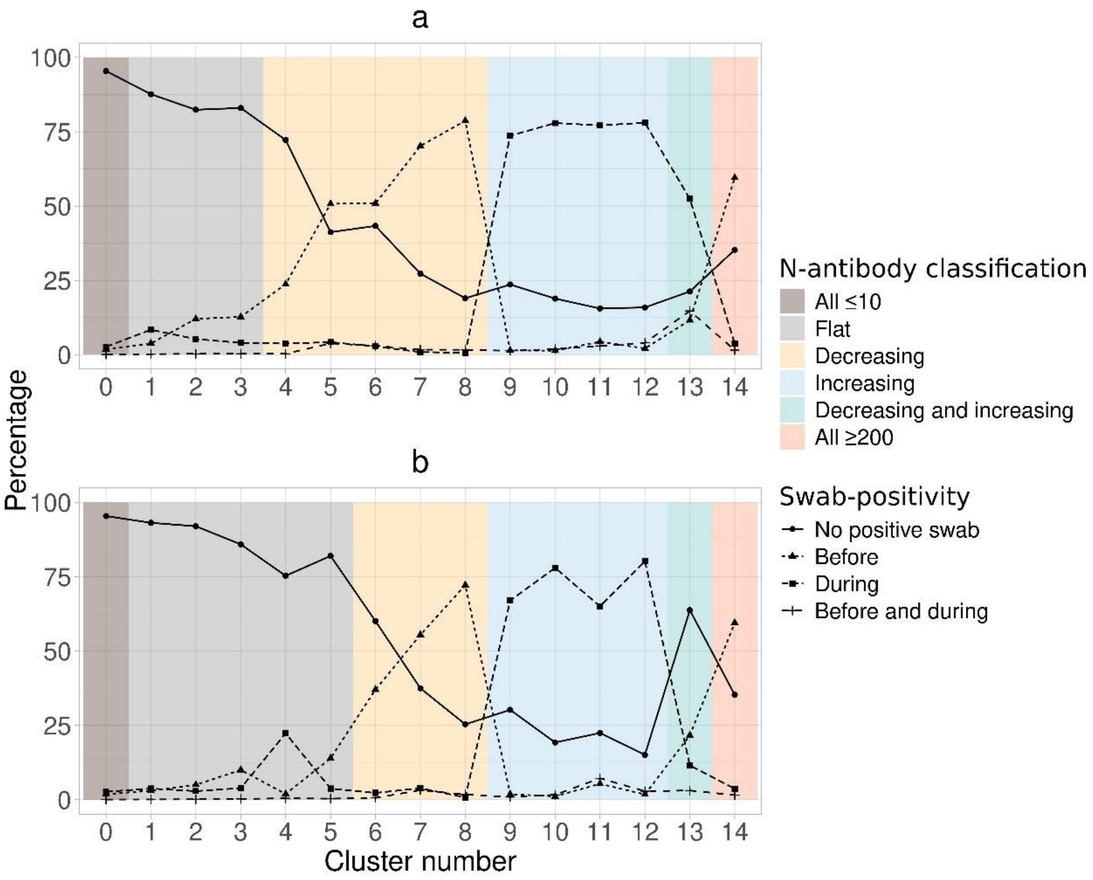

Supplementary Figure 5. *Percentages of each swab-positive infection group in each cluster, with (a) identity transformations, (b) log2 transformation.* Cluster numbers are non-informative and correspond to the cluster numbers in Supplementary Figure 3. N-antibody classifications in (a) cluster 0: All N-antibody levels ≤10, clusters 1-3: flat, clusters 4-8: decreasing, clusters 9-12: increasing, cluster 13: decreasing and increasing, and cluster 14: all N-antibody levels ≥ 200; (b) cluster 0: all N-antibody levels ≤10, clusters 1-5: flat, clusters 6-8: decreasing, clusters 9-12: increasing, cluster 13: decreasing and increasing, cluster 14: all N-antibody levels≥ 200. Note that the same participants may be classified in different clusters for the two different transformations.

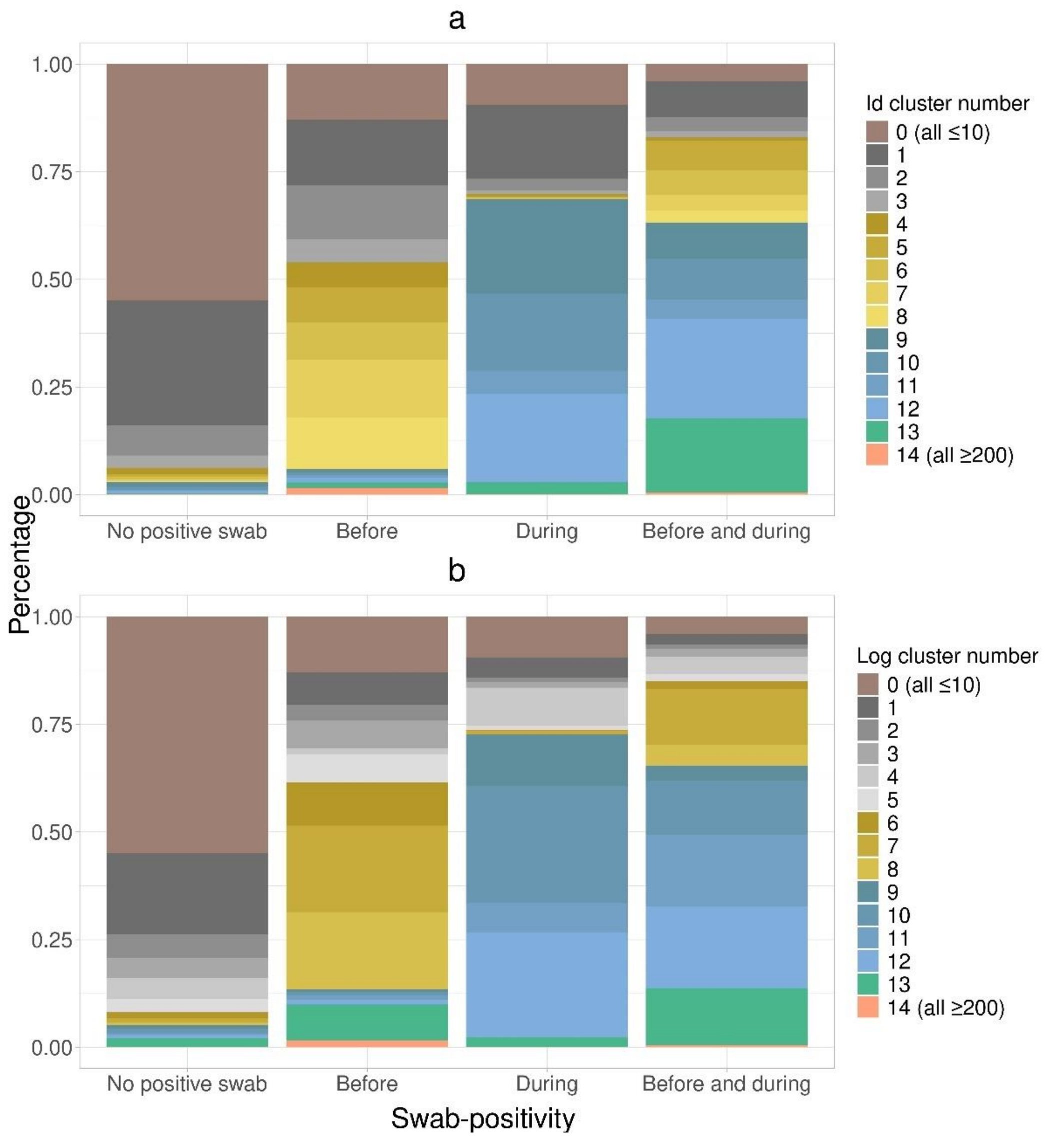

Supplementary Figure 6. *Confusion matrix and trajectories for the N-antibody id and log2 clustering classifications.* (a) confusion matrix with counts per (dis)concordant group of the id and log_2 clustering classifications. The colour intensity of the tiles indicates the total percentage. (b) N-antibody trajectories for the (dis)concordant groups with the id and log_2 classifications. Each frame contains a random sample of 200 N-antibody trajectories, or all trajectories where there were <200 participants available. For comparability, trajectories are centered on the midpoint between the maximum difference between any two consecutive measurements per participant. Black line depicts a generalised additive modelling smooth for all N-antibody measurements assayed between the 10th and 90th percentile of the centered days in each frame.

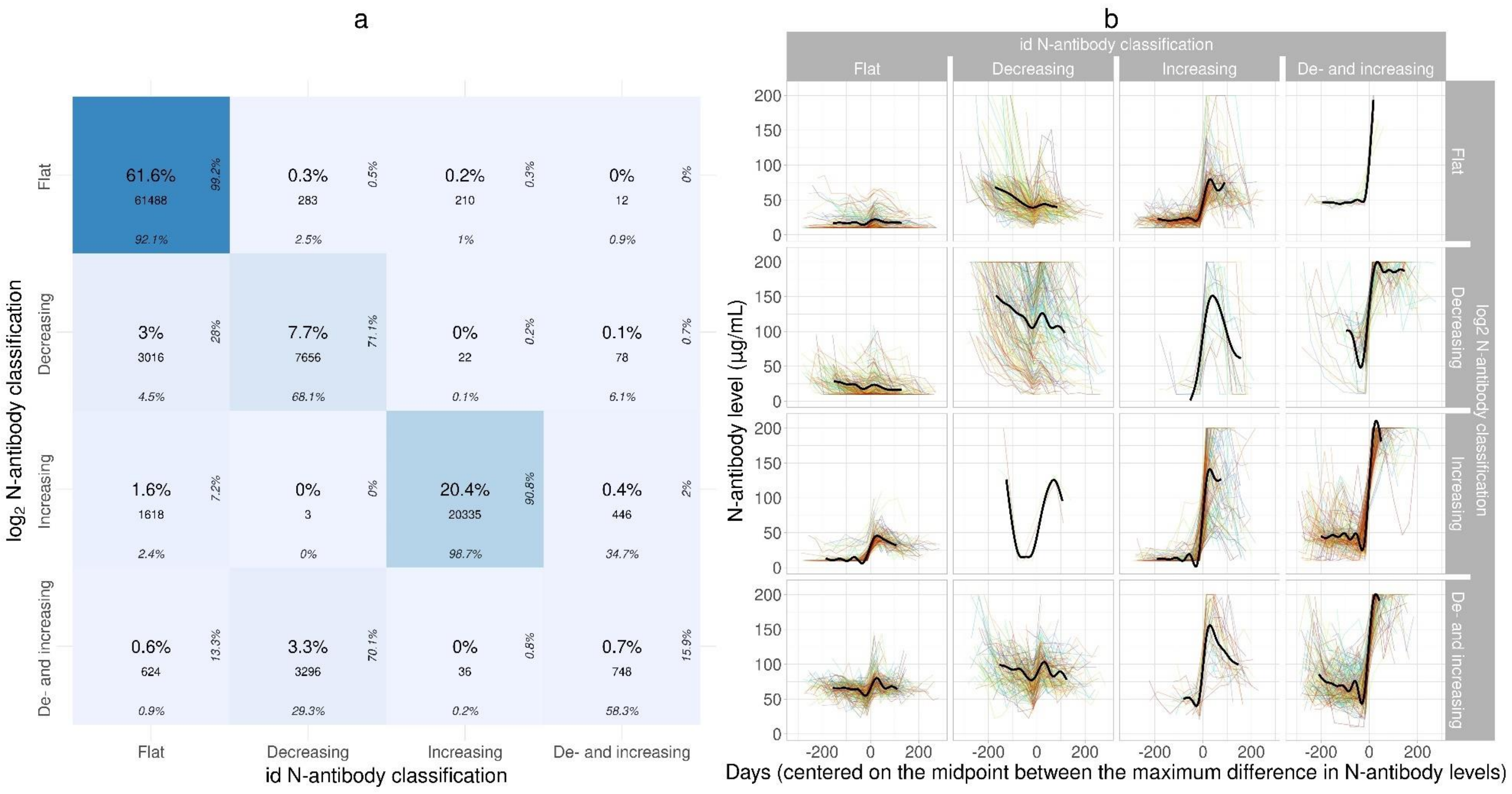

Supplementary Figure 7. *Reclassified N-antibody trajectories.*
Where id and log_2 classification differed (N=9,644), participants were classified using visualization, as shown below. For comparability, trajectories are centered on the midpoint between the maximum difference between any two consecutive measurements per participant. Black line depicts a generalised additive modelling smooth for all N-antibody measurements assayed between the 10th and 90th percentile of the centered days in each frame. The black line is only depicted if a frame contains N-antibody trajectories for more than 10 individuals. Interestingly, the N-antibody trajectories for 54 participants in cluster 13 using identity clustering and cluster 10 using log2 transformed clustering implies two different infections during the participant's study period.

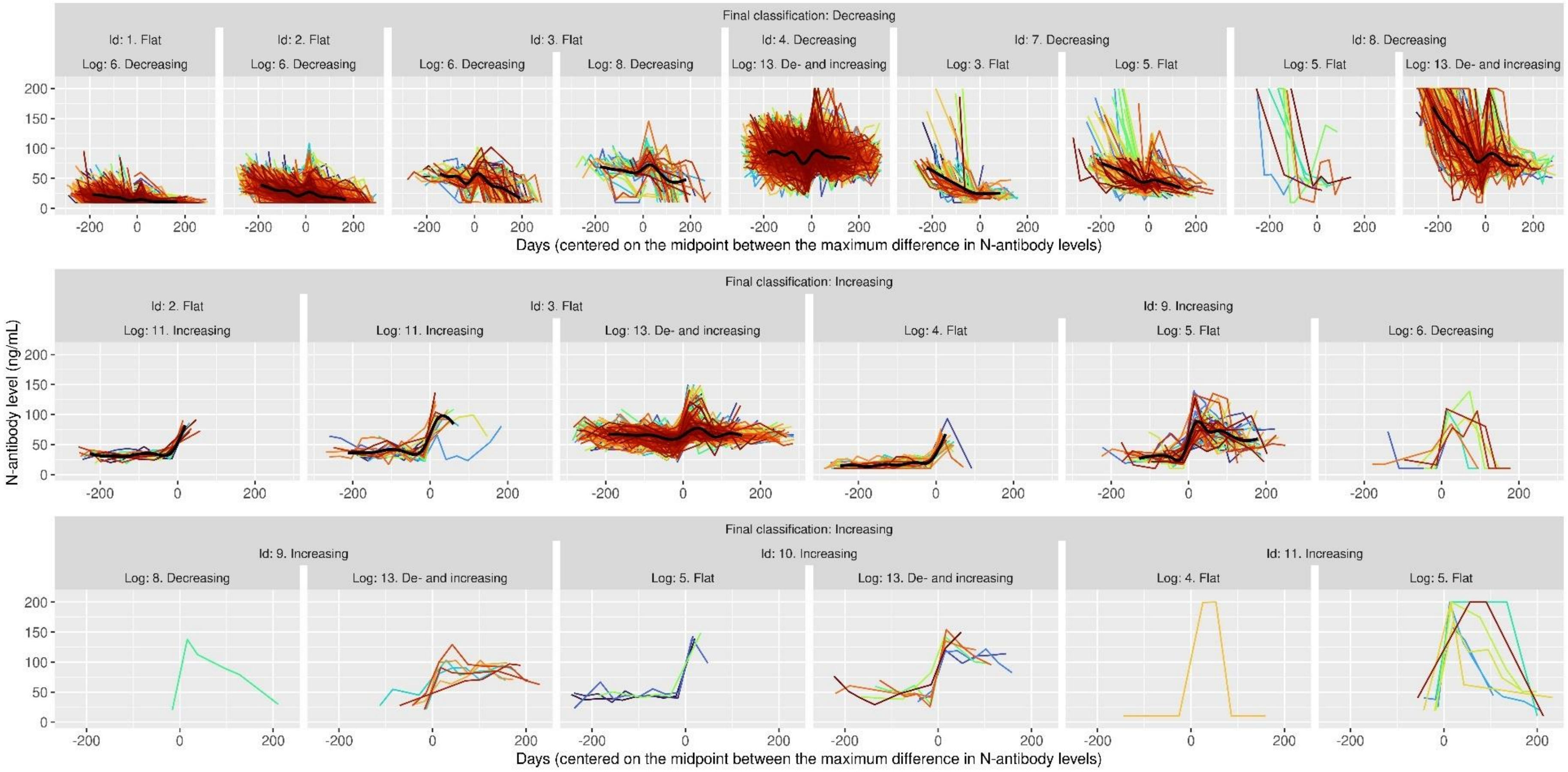

(continued)

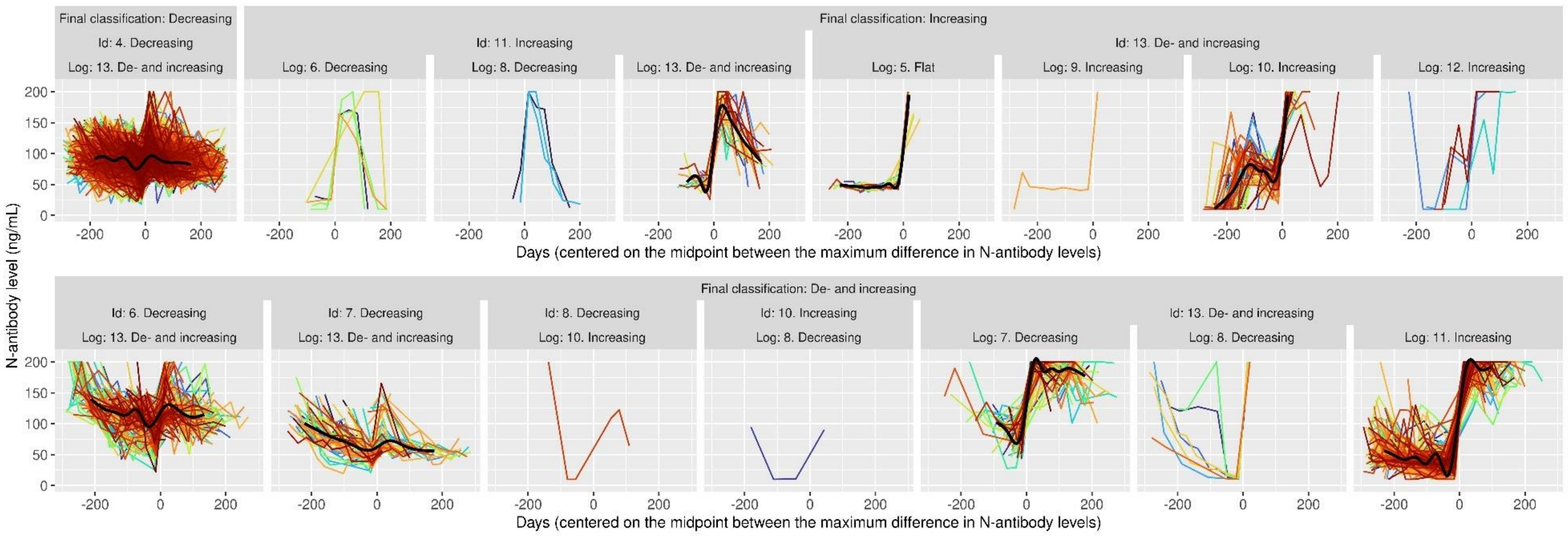
Final classification: Decreasing
Id: 4. Decreasing
Log: 13. De- and increasing
Final classification: Increasing
Id: 11. Increasing
Log: 6. Decreasing
Log: 8. Decreasing
Log: 13. De- and increasing
Log: 5. Flat
Id: 13. De- and increasing
Log: 9. Increasing
Log: 10. Increasing
Log: 12. Increasing
N-antibody level (ng/mL)
Days (centered on the midpoint between the maximum difference in N-antibody levels)
Final classification: De- and increasing
Id: 6. Decreasing
Log: 13. De- and increasing
Id: 7. Decreasing
Log: 13. De- and increasing
Id: 8. Decreasing
Log: 10. Increasing
Id: 10. Increasing
Log: 8. Decreasing
Id: 13. De- and increasing
Log: 7. Decreasing
Log: 8. Decreasing
Log: 11. Increasing
Days (centered on the midpoint between the maximum difference in N-antibody levels)

Supplementary Figure 8. *Distribution of days between the N-antibody (hypothetical) infection date and closest swab-positive infection.*

Note: For N-antibody (hypothetical) infections, infection date was estimated as 14 days before the midpoint between the two measurements with the greatest increase between them. The figure includes all 17,623 individuals with ≥4 N-antibody measurements, an increasing or de- and increasing N-antibody trajectory (identified with the clustering) and a swab-positive infection during their study period. Excluding 505 participants with estimated infection date ≥60 days from the swab-positive infection for visualization. The box represents the inter quartile range, while the middle line represents the median. The whiskers represent a distance of 1.5 times the interquartile range starting from the first and third quartile. Dots represent points that fall outside the whiskers (outliers).

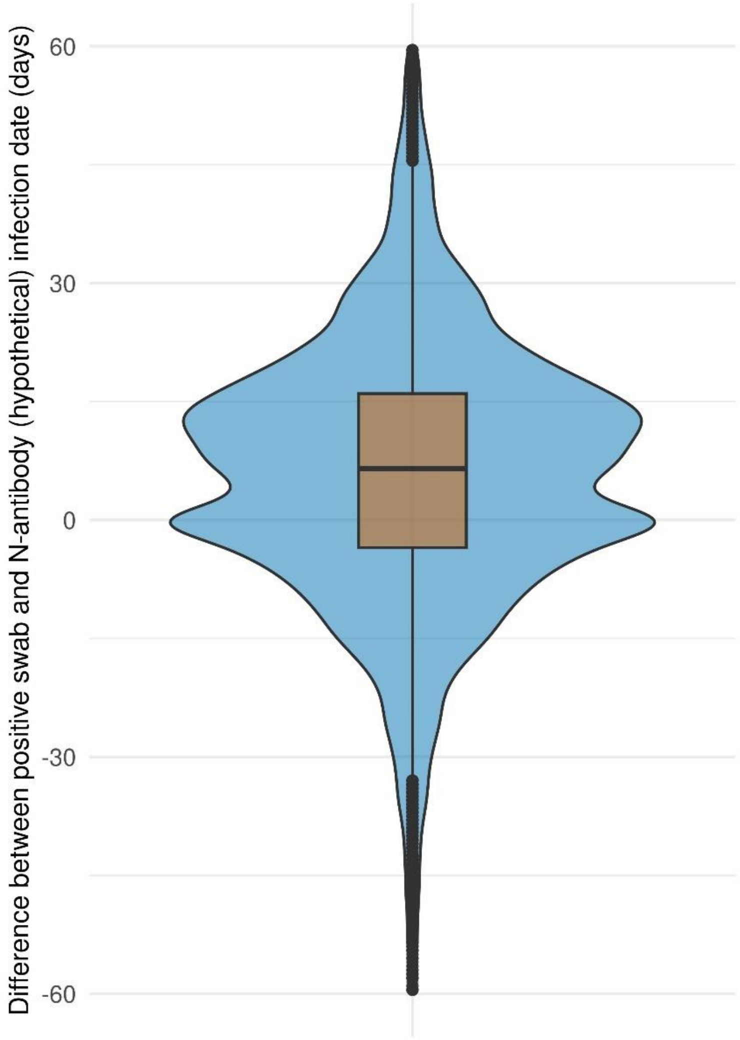

Supplementary Figure 9. *Flow chart for logistic regression.*
Note: Ct values only available for swab-positive infections identified from PCR tests done as part of the COVID-19 Infection Survey, or at the same laboratories using the same PCR test as part of the national testing programme in England and Wales (see Methods)

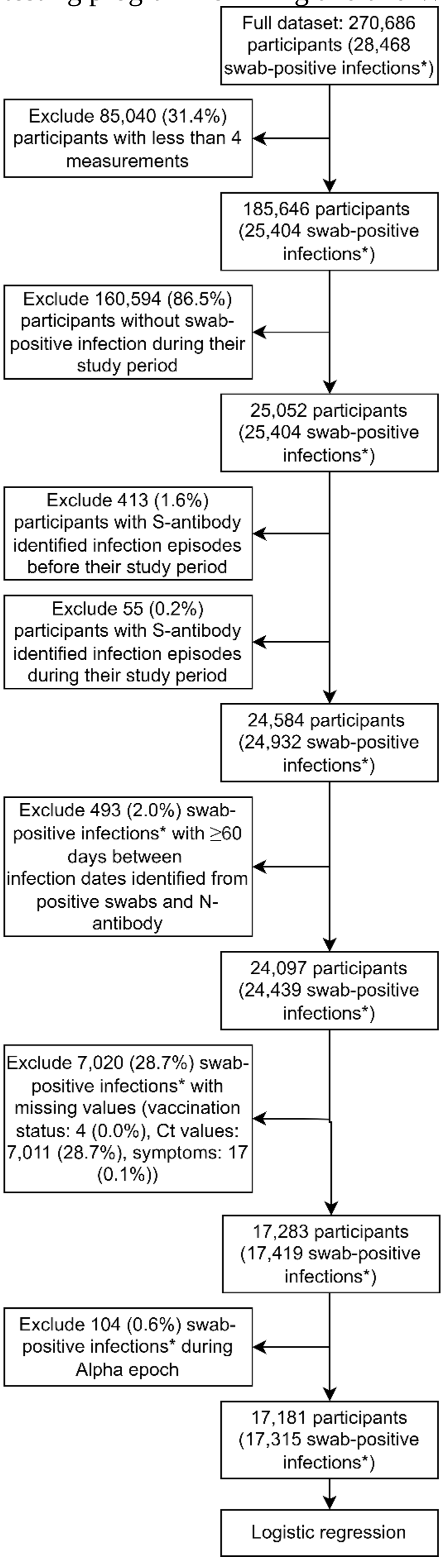

*swab-positive infections during the study period

Supplementary Figure 10. *Visualisation of N-antibody trajectories by trajectory-based and threshold-based N-antibody classification*

Trajectory-based and threshold-based positivity, stratified by swab positivity (all during the participant's study period). Each frame contains a random sample of 200 N-antibody trajectories. Black line depicts a generalised additive modelling smooth for all N-antibody measurements assayed between the 10th and 90th percentile of the centered days in each frame. Counts refer to the total number of N-antibody trajectories in each frame. Trajectory-based seronegative participants have either *decreasing* or *flat* N-antibody trajectories and seropositives are either *increasing* or *de- and increasing*. Threshold-based seronegative participants have either all N-antibody measurements below the manufacturer's threshold of 30 ng/mL or the first N-antibody measurement above 30ng/mL and no increase from below 30 ng/mL to above 30 ng/mL during the participant's study period. Threshold-based seropositives have an increase from below 30 ng/mL to above 30 ng/mL during the participant's study period

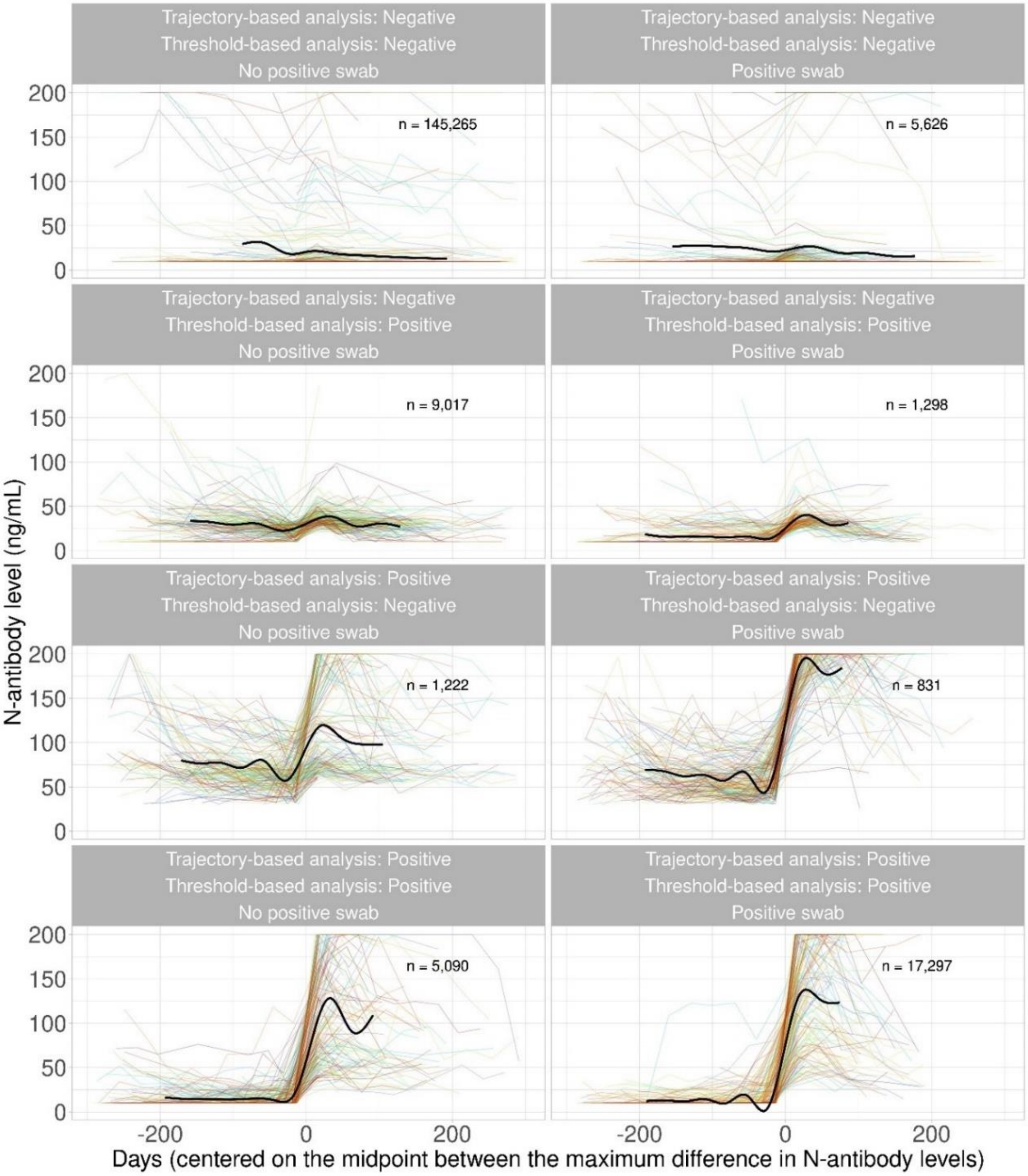

Supplementary Figure 11. *Visualisation of N-antibody trajectories by trajectory-based and main fourfold-based classification*

Trajectory-based and fourfold-based positivity, stratified by swab positivity (all during the participant's study period). Each frame contains a random sample of maximal 200 N-antibody trajectories. Black line depicts a generalised additive modelling smooth for all N-antibody measurements assayed between the 10th and 90th percentile of the centered days in each frame. Counts refer to the total number of N-antibody trajectories in each frame. Trajectory-based seronegative participants have either *decreasing* or *flat* N-antibody trajectories and seropositives are either *increasing* or *de- and increasing*. Fourfold-based seronegative participants have no fourfold increase in consecutive antibody measurements and fourfold-based seropositives do have such an increase.

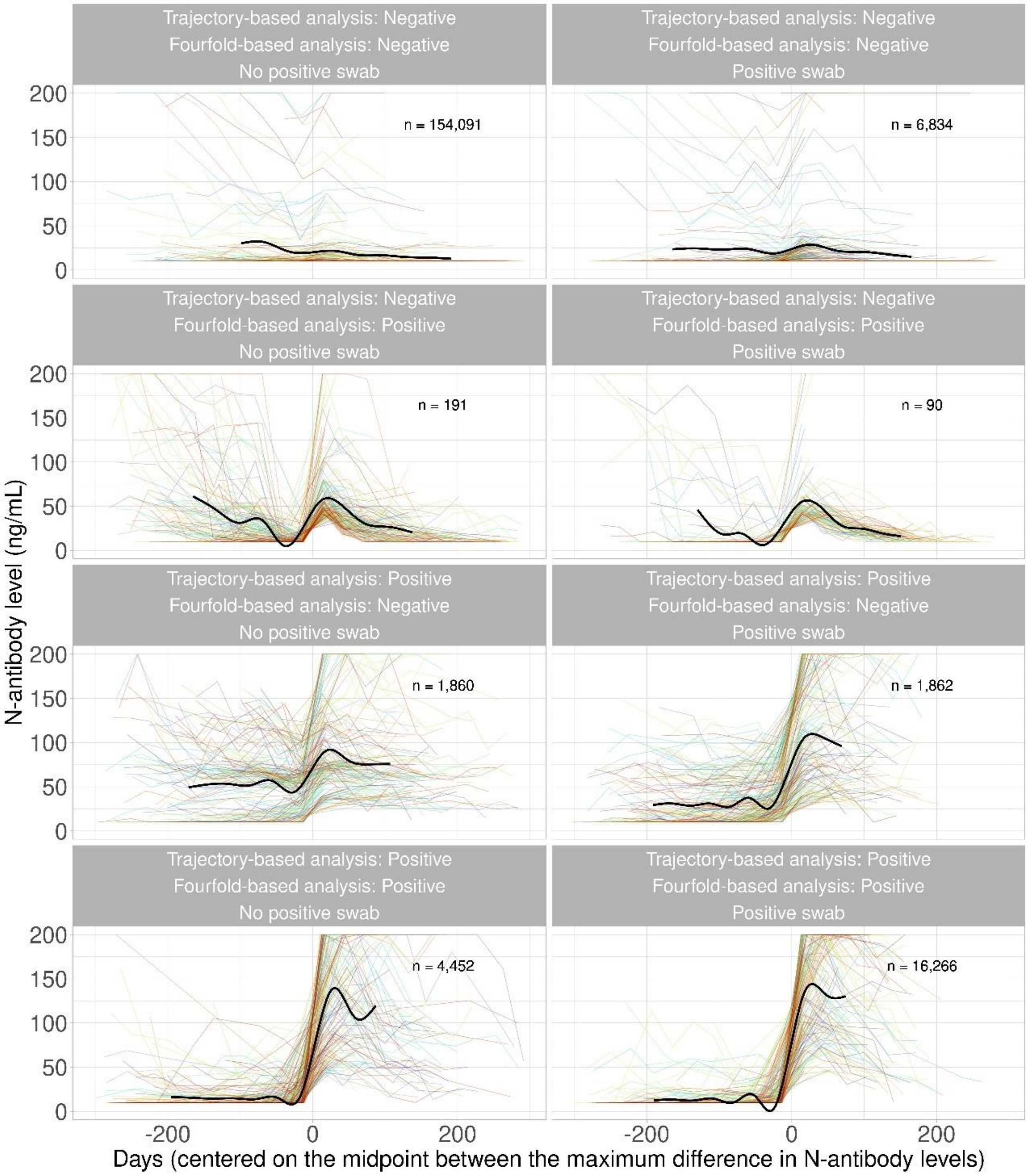

Supplementary Figure 12. *Visualisation of N-antibody trajectories by trajectory-based and fourfold-based classification (sensitivity analysis)*

Trajectory-based and fourfold-based positivity sensitivity analysis, stratified by swab positivity (all during the participant's study period). Each frame contains a random sample of maximal 200 N-antibody trajectories. Black line depicts a generalised additive modelling smooth for all N-antibody measurements assayed between the 10th and 90th percentile of the centered days in each frame. Counts refer to the total number of N-antibody trajectories in each frame. Trajectory-based seronegative participants have either *decreasing* or *flat* N-antibody trajectories and seropositives are either *increasing* or *de- and increasing*. Fourfold-based (sensitivity) seronegative participants have no fourfold increase in consecutive antibody measurements, counting increases to ≥200 ng/mL as a fourfold increase regardless of starting value.

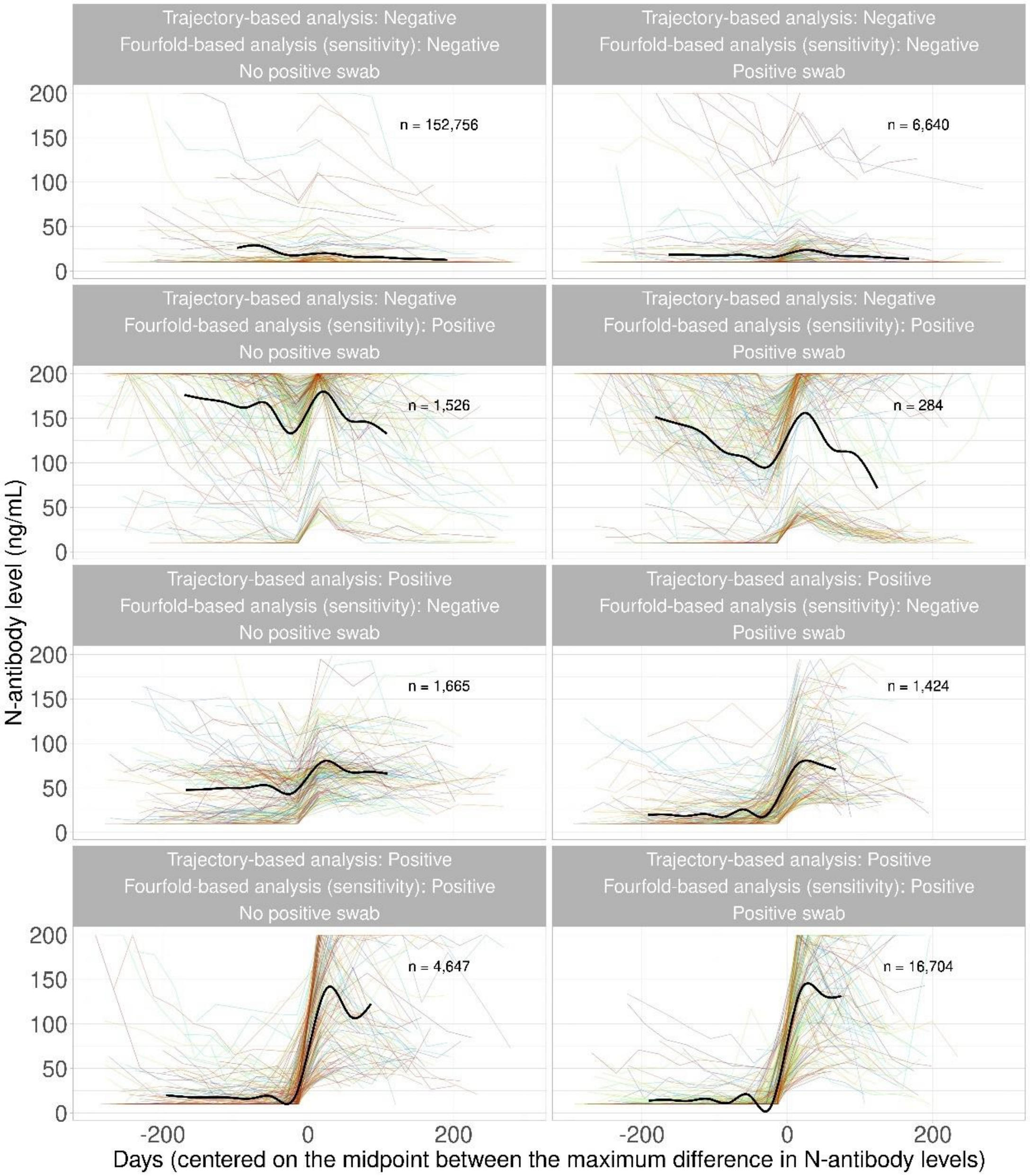

References

1. Pouwels, K.B., *et al.* Community prevalence of SARS-CoV-2 in England from April to November, 2020: results from the ONS Coronavirus Infection Survey. *Lancet Public Health* **6**, e30-e38 (2021).
2. Tavenard, R., *et al.* Tslearn, A Machine Learning Toolkit for Time Series Data. *J Mach Learn Res* **21** 1-6 (2020).
3. Petitjean, F., Ketterlin, A. & Gançarski, P. A global averaging method for dynamic time warping, with applications to clustering. *Pattern Recogn* **44**, 678-693 (2011).
4. Chabchoub, Y. & Fricker, C. Classification of the Velib Stations Using Kmeans, Dynamic Time Wraping and Dba Averaging Method. *2014 International Workshop on Computational Intelligence for Multimedia Understanding (Iwcim)* (2014).
5. Arthur, D. & Vassilvitskii, S. k-means plus plus : The Advantages of Careful Seeding. *Proceedings of the Eighteenth Annual Acm-Siam Symposium on Discrete Algorithms*, 1027-1035 (2007).
