# Supplementary material for: Identification of undetected SARS-CoV-2 infections by clustering of Nucleocapsid antibody trajectories
Source: Nat Commun. 2025 May 14;16:4466. doi: 10.1038/s41467-025-57370-z (PMC12078723; doi:10.1038/s41467-025-57370-z)
Supplement: Supplementary file 2 — Reporting Summary [file 41467_2025_57370_MOESM2_ESM.pdf]

nature portfolio

Corresponding author(s): Leslie R. Zwerwer
Last updated by author(s): 17-02-2025

Reporting Summary

Nature Portfolio wishes to improve the reproducibility of the work that we publish. This form provides structure for consistency and transparency in reporting. For further information on Nature Portfolio policies, see our Editorial Policies and the Editorial Policy Checklist.

Statistics

For all statistical analyses, confirm that the following items are present in the figure legend, table legend, main text, or Methods section.

n/a	Confirmed	
☐	☒	The exact sample size (*n*) for each experimental group/condition, given as a discrete number and unit of measurement
☐	☒	A statement on whether measurements were taken from distinct samples or whether the same sample was measured repeatedly
☐	☒	The statistical test(s) used AND whether they are one- or two-sided<br>*Only common tests should be described solely by name; describe more complex techniques in the Methods section.*
☐	☒	A description of all covariates tested
☐	☒	A description of any assumptions or corrections, such as tests of normality and adjustment for multiple comparisons
☐	☒	A full description of the statistical parameters including central tendency (e.g. means) or other basic estimates (e.g. regression coefficient) AND variation (e.g. standard deviation) or associated estimates of uncertainty (e.g. confidence intervals)
☐	☒	For null hypothesis testing, the test statistic (e.g. *F*, *t*, *r*) with confidence intervals, effect sizes, degrees of freedom and *P* value noted<br>*Give P values as exact values whenever suitable.*
☒	☐	For Bayesian analysis, information on the choice of priors and Markov chain Monte Carlo settings
☒	☐	For hierarchical and complex designs, identification of the appropriate level for tests and full reporting of outcomes
☐	☒	Estimates of effect sizes (e.g. Cohen's *d*, Pearson's *r*), indicating how they were calculated

Our web collection on statistics for biologists contains articles on many of the points above.

Software and code

Policy information about availability of computer code

Data collection	De-identified data were prepared for data analysis using Stata MP 16.
Data analysis	The clustering analysis was performed in Python version 3.10.12 (jupyter-notebook version 7.1.2) using the packages pandas (version 1.4.2), numpy (version 1.23.5), tslearn (version 0.5.2) and dill (version 0.3.4). All other analyses were performed in R version 4.3 using the packages tidyverse (version 2.0.0), ggplot2 (version 3.5.0), dplyr (version 1.1.4), Rcapture (version 1.4.4), nnet (version 7.3.19) and splines (version 4.3.2). A copy of the analysis code is available at: https://github.com/UMCG-Global-Health/COVID-19_N-antibodies (https://doi.org/10.5281/zenodo.13934702).

For manuscripts utilizing custom algorithms or software that are central to the research but not yet described in published literature, software must be made available to editors and reviewers. We strongly encourage code deposition in a community repository (e.g. GitHub). See the Nature Portfolio guidelines for submitting code & software for further information.

Data

Policy information about availability of data

All manuscripts must include a data availability statement. This statement should provide the following information, where applicable:

- Accession codes, unique identifiers, or web links for publicly available datasets
- A description of any restrictions on data availability
- For clinical datasets or third party data, please ensure that the statement adheres to our policy

De-identified study data are available for access by accredited researchers in the ONS Secure Research Service (SRS) for accredited research purposes under part 5, chapter 5 of the Digital Economy Act 2017. Individuals can apply to be an accredited researcher using the short form on https://researchaccreditationservice.ons.gov.uk/ons/ONS_registration.ofml. Accreditation requires completion of a short free course on accessing the SRS. To request access to data in the SRS, researchers must submit a research project application for accreditation in the Research Accreditation Service (RAS). Research project applications are considered by the project team and the Research Accreditation Panel (RAP) established by the UK Statistics Authority at regular meetings. Project application example guidance and an exemplar of a research project application are available. A complete record of accredited researchers and their projects is published on the UK Statistics Authority website to ensure transparency of access to research data. For further information about accreditation, contact Research.Support@ons.gov.uk or visit the SRS website.

Research involving human participants, their data, or biological material

Policy information about studies with human participants or human data. See also policy information about sex, gender (identity/presentation), and sexual orientation and race, ethnicity and racism.

Reporting on sex and gender	The COVID-19 infection survey invited all individuals aged 2 years or older living within randomly selected private households across the UK to participate (regardless of sex). Sex was self-reported by participants (i.e. female/male). The sample adequately represented both sexes. Demographics on sex were reported and sex was included as a covariate in the logistic regression to understand the association between sex and N-antibody non-response (vs. response) in swab-positive infections. Given the extensive findings in the current study, we did not conduct further sex-based analyses.
Reporting on race, ethnicity, or other socially relevant groupings	Ethnicity was self-reported by participants (white vs. non-white). White participants were overrepresented. Demographics on ethnicity were reported and ethnicity was included as a covariate in the logistic regression to understand the association between ethnicity and N-antibody non-response (vs. response) in swab-positive infections.
Population characteristics	All characteristics of the full population can be found in the manuscript (Result; Population) and Table 1. Characteristics of the clustering population can be found in Supplementary Table 1.
Recruitment	The COVID-19 infection survey invited all individuals aged 2 years or older living within randomly selected private households across the UK to participate. Initially, those aged ≥16 years from a random 10-20% households were asked for optional consent to give monthly venous blood samples for serological testing; this was expanded to a larger randomly selected subgroup of households from April 2021 using capillary blood sampling to examine vaccine responses (prioritising those with longer survey participation). Moreover, any participant ≥16 years testing PCR-positive through December 2021 was invited to provide blood samples on their subsequent monthly follow up visits.<br><br>To optimize response rates participants received a 50£ voucher for completing the enrollment visit and a 25£ voucher for each subsequent visit (see also: https://www.ons.gov.uk/surveys/informationforhouseholdsandindividuals/householdandindividualsurveys/covid19infectionsurveycis/howtotakepart). This has led to a broadly representative sample in terms of country, sex and age (see also: https://www.ons.gov.uk/peoplepopulationandcommunity/healthandsocialcare/conditionsanddiseases/methodologies/coronaviruscovid19infectionsurveyqmi). However, participants of non-white ethnicity and in one or two person households are slightly underrepresented. Moreover despite the generous compensation, the survey data may suffer from bias due to non-response, although the degree of this is unknown.
Ethics oversight	Following verbal consent, study workers visited each household, and recruited all residents aged 2 years or older who provided written informed consent (from parents/carers for those under 16 years; those aged 10-15 years also provided written assent). Participants could also provide optional consent for subsequent weekly visits in the first month and then monthly, up to the earliest of March 2023, when they became no longer resident at the selected address or no longer wished to participate (98% consented to post-enrolment visits). Ethical approval was obtained from the South Central Berkshire B Research Ethics Committee (20/SC/0195).

Note that full information on the approval of the study protocol must also be provided in the manuscript.

Field-specific reporting

Please select the one below that is the best fit for your research. If you are not sure, read the appropriate sections before making your selection.

☒ Life sciences ☐ Behavioural & social sciences ☐ Ecological, evolutionary & environmental sciences

For a reference copy of the document with all sections, see nature.com/documents/nr-reporting-summary-flat.pdf

Life sciences study design

All studies must disclose on these points even when the disclosure is negative.

Sample size

270,686 participants provided blood samples for serological testing in the period when N-antibodies were assayed within the COVID-19 Infection Survey (February 28, 2021 - January 30, 2022). 185,646 were included in the analysis which was restricted to those with 4 or more N-antibody measurements in whom clustering could be performed. There was no sample size calculation performed specifically for N-antibody measurements or this study.
The primary determination of the numbers providing blood samples was based on monitoring S-antibody positivity. The total blood target of ~125,500 individuals sampled every month in England for approximately one year from March 2021 through to April 2022 was designed to provide estimates of S-antibody immunity within different regions (~10,000-15,000 per month) with a margin of error below 2%, and within different age groups over 16 years within regions (~2,000 per month) with margins of error below 5%. N-antibody was assayed in parallel on these samples.

The final analysis sample size of over 185,646 provides high precision around our estimates of missed infections, as stated in the manuscript.

Data exclusions

We classified different types of N-antibody trajectories in participants with ≥4 N-antibody measurements, in order to ensure the N-antibody trajectories had sufficient information to detect SARS-CoV-2 infections. This excluded 85,040 participants (see also Supplementary Figure 2). This sample was used for the main analyses.

We also investigated lack of N-antibody seroconversion amongst participants with swab-positive infections and ≥4 N-antibody measurements (exclusion of 245,634 participants). For this analysis we further excluded participants with a S-antibody identified infection (as this could possibly be a marker of (previous) unregistered vaccination), participants with 60 days or more between the estimated N-antibody hypothetical infection date and swab-positive date, all participants with missing values and a very small number of infections before the emergence of Delta (exclusion of in total 7,871 participants). Details for this analysis can be found in the Methods section (Associations with participant characteristics) and Supplementary Figure 9.

Replication

Taking into account the millions of assays performed for this study and the associated financial constraints, all measurements and assays were conducted once.

Randomization

Random recruitment: Data came from the UK's Office for National Statistics (ONS) COVID-19 Infection Survey (ISRCTN21086382, protocol on https://www.ndm.ox.ac.uk/covid-19/covid-19-infection-survey/protocol-and-information-sheets). The COVID-19 infection survey continuously selected private households from address lists and prior surveys conducted by the ONS or the Northern Ireland Statistics and Research Agency, to collect a representative sample across the UK (i.e. England, Wales, Northern Ireland, and Scotland).

Blinding

Not applicable since no intervention was used. This was a retrospective observational study.

Reporting for specific materials, systems and methods

We require information from authors about some types of materials, experimental systems and methods used in many studies. Here, indicate whether each material, system or method listed is relevant to your study. If you are not sure if a list item applies to your research, read the appropriate section before selecting a response.

Materials & experimental systems

n/a	Involved in the study	
☐	☒	Antibodies
☒	☐	Eukaryotic cell lines
☒	☐	Palaeontology and archaeology
☒	☐	Animals and other organisms
☐	☒	Clinical data
☒	☐	Dual use research of concern
☒	☐	Plants

Methods

n/a	Involved in the study	
☒	☐	ChIP-seq
☒	☐	Flow cytometry
☒	☐	MRI-based neuroimaging

Antibodies

Antibodies used

Levels of SARS-CoV-2 S-antibody and N-antibody were tested on venous or capillary blood samples using an enzyme-linked immunosorbent assay (ELISA) detecting anti-trimeric spike and nucleocapsid IgG developed by the University of Oxford. Before 26 February 2021, the S-antibody assay used fluorescence detection, with a positivity threshold of 8 million units validated on banks of known SARS-CoV-2-positive and -negative samples. After this, the S-antibody used a commercialized CE-marked version of the assay, the Thermo Fisher OmniPATH 384 Combi SARS-CoV-2 IgG ELISA (Thermo Fisher Scientific), with the same antigen and colorimetric detection, reporting normalized results in ng/mL of mAb45 monoclonal antibody equivalents and using 42ng/mL as the threshold for an IgG-positive or -negative result (corresponding to the 8 million units with fluorescence detection). SARS-CoV-2 N-antibody levels were tested using a research-use only assay.

Validation

Detailed information on the validation of the Thermo Fisher OmniPATH 384 Combi SARS-CoV-2 IgG ELISA kit can be found in the manufacturer's instructions for use. In previous research this assay had a sensitivity of 99·1% (97·8–99·7) and specificity of 99·0%

(98·1–99·5) (doi: 10.1016/S1473-3099(20)30634-4).
The performance report of the N-antibody assay is available at: https://www.ndm.ox.ac.uk/covid-19/covid-19-infection-survey/n-antibody-assay-performance.

Clinical data

Policy information about clinical studies

All manuscripts should comply with the ICMJE guidelines for publication of clinical research and a completed CONSORT checklist must be included with all submissions.

Clinical trial registration: ISRCTN21086382

Study protocol: https://www.ndm.ox.ac.uk/covid-19/covid-19-infection-survey/protocol-and-information-sheets

Data collection: Data came from the UK's Office for National Statistics (ONS) COVID-19 Infection Survey. The data used for this study were collected between April 26, 2020 and January 30, 2022. The study period was defined as the period in which participants had N-antibody measurements available. All survey data after the participant's study period was excluded from the analysis.

Outcomes: SARS-CoV-2 N-antibody levels were tested using a research-use only assay. For participants with 4 or more N-antibody measurements we assessed N-antibody seropositivity by clustering the N-antibody trajectories and arbitrarily classifiying them based on expected trajectories following infection (Supplementary Fig.3). Then using the number of swab-positives and N-antibody trajectory-based positives in a method-dependent capture-recapture model we assessed how many infections remained undetected by either methods.

Secondary analyses included:
1. Sensitivity analyses on the number of undectected infections using a method-dependent capture-recapture model (i.e. subgroup analyses for different vaccination statuses and variants, reclassifications of those with more than 60 days between swab-positive date and hypothetical N-antibody infection date, N-antibody seropositivity based on a threshold of 30 ng/mL, N-antibody seropositivity based on a fourfold-increase and a sensitivity analysis on the fourfold-based N-antibody classifications).
2. Estimating a hypothetical N-antibody infection date and comparing this date to the swab-positive infection date.
3. Examining the associations between characteristics (i.e. age, sex, ethnicity, healthcare worker, long-term health condition, vaccination status, cycle threshold values, symptoms and variant) and N-antibody non-response (vs. response) in swab-positive infections.
4. Comparisons of N-antibody (hypothetical) infections with infections defined using different data sources.

Plants

Seed stocks: NA

Novel plant genotypes: NA

Authentication: NA
